# (2*E*)-3-(2-Chloro-6-methyl-3-quinol­yl)-1-(1-naphth­yl)prop-2-en-1-one

**DOI:** 10.1107/S1600536810007828

**Published:** 2010-03-06

**Authors:** Syed Umar Farooq Rizvi, Hamid Latif Siddiqui, Muhammad Zia-ur-Rehman, Muhammad Azam, Masood Parvez

**Affiliations:** a Institute of Chemistry, University of the Punjab, Lahore 54590, Pakistan; b Applied Chemistry Research Centre, PCSIR Laboratories Complex, Lahore 54600, Pakistan; c Institute of Biochemistry, University of Balochistan, Quetta 8700, Pakistan; d Department of Chemistry, The University of Calgary, 2500 University Drive NW, Calgary, Alberta, Canada T2N 1N4

## Abstract

In the title mol­ecule, C_23_H_16_ClNO, the quinoline and naphthalene ring systems are individually planar, with maximum deviations of 0.020 (2) and 0.033 (2) Å, respectively, and are inclined at a dihedral angle of 30.01 (4)°. Intra­molecular C—H⋯O and C—H⋯Cl inter­actions occur. The crystal structure is devoid of any classical hydrogen bonds, but symmetry-related mol­ecules are linked *via* weak C—H⋯Cl inter­actions, forming chains propagating in [001].

## Related literature

For background literature on chalcones, see: Drexler & Amiridis (2003 ▶); Opletalova & Sedivy (1999 ▶); Oyedapo *et al.* (2004 ▶); Prabhavat & Ghiya (1998 ▶); Varga *et al.* (2003 ▶). For bond distances, see: Allen (2002 ▶). For the preparation of 2-chloro-6-methyl-3-formyl­quinoline, see: Meth-Cohn *et al.* (1981 ▶).
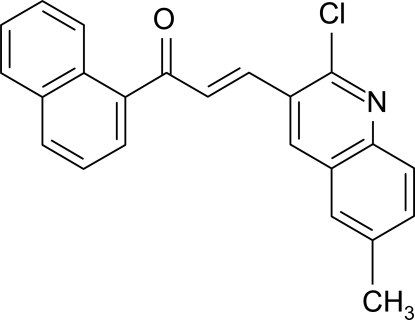


## Experimental

### 

#### Crystal data

C_23_H_16_ClNO
*M* *_r_* = 357.82
Monoclinic, 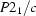

*a* = 16.919 (8) Å
*b* = 7.146 (3) Å
*c* = 14.829 (5) Å
β = 103.29 (2)°
*V* = 1744.9 (13) Å^3^
*Z* = 4
Mo *K*α radiation
μ = 0.23 mm^−1^
*T* = 173 K
0.14 × 0.12 × 0.05 mm

#### Data collection

Nonius KappaCCD diffractometer
Absorption correction: multi-scan (*SORTAV*; Blessing, 1997 ▶) *T*_min_ = 0.968, *T*_max_ = 0.989
6886 measured reflections
3988 independent reflections
2575 reflections with *I* > 2σ(*I*)
*R*_int_ = 0.039

#### Refinement

*R*[*F* ^2^ > 2σ(*F* ^2^)] = 0.051
*wR*(*F* ^2^) = 0.131
*S* = 1.01
3988 reflections
236 parameters
H-atom parameters constrained
Δρ_max_ = 0.24 e Å^−3^
Δρ_min_ = −0.29 e Å^−3^

### 

Data collection: *COLLECT* (Hooft, 1998 ▶); cell refinement: *DENZO* (Otwinowski & Minor, 1997 ▶); data reduction: *SCALEPACK* (Otwinowski & Minor, 1997 ▶); program(s) used to solve structure: *SHELXS97* (Sheldrick, 2008 ▶); program(s) used to refine structure: *SHELXL97* (Sheldrick, 2008 ▶); molecular graphics: *ORTEP-3 for Windows* (Farrugia, 1997 ▶); software used to prepare material for publication: *SHELXL97*.

## Supplementary Material

Crystal structure: contains datablocks global, I. DOI: 10.1107/S1600536810007828/su2165sup1.cif

Structure factors: contains datablocks I. DOI: 10.1107/S1600536810007828/su2165Isup2.hkl

Additional supplementary materials:  crystallographic information; 3D view; checkCIF report

## Figures and Tables

**Table 1:** Hydrogen-bond geometry (Å, °)

*D*—H⋯*A*	*D*—H	H⋯*A*	*D*⋯*A*	*D*—H⋯*A*
C7—H7⋯Cl1^i^	0.95	2.86	3.792 (2)	166
C11—H11⋯Cl1	0.95	2.65	3.045 (2)	106
C11—H11⋯O1	0.95	2.45	2.788 (3)	101
C22—H22⋯O1	0.95	2.33	2.924 (3)	120

Symmetry code: (i) 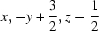.

**Fig. 1.**
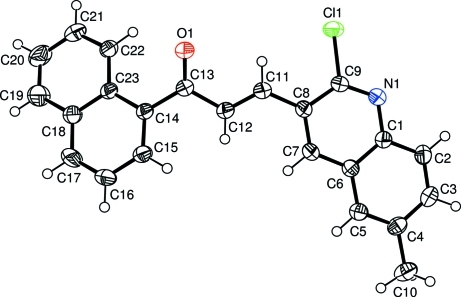
ORTEP-3 (Farrugia, 1997) drawing of the title molecule, with displacement ellipsoids drawn at the 50% probability level.

**Fig. 2.**
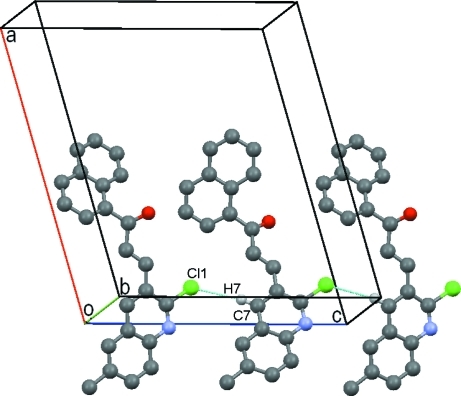
A view along the b-axis of the crystal packing of the title compound, showing the C—H···Cl interactions as dashed lines [see Table 1 for details; H-atoms not involved in hydrogen bonds have been omitted for clarity].

## supplementary crystallographic information

### Comment

1,3-Diaryl-2-propen-1-ones, commonly known as chalcones, are normally synthesized by Claisen-Schmidt condensation (Oyedapo *et al.*, 2004). They are used as precursors to synthesize many heterocyclic compounds like flavonoids (Drexler & Amiridis, 2003), pyrimidines, imidazoles (Varga *et al.*, 2003) etc. Chalcones have already been recognized as anti-bacterial, anti-tuberculous, anti-tumor, anti-inflammatory, anti-viral, anti-microbial and anti-protozoal gastroprotective agents (Opletalova & Sedivy, 1999). They may also be converted to 2-mercaptopyrimidines, which have anti-cancer, anti-tubercular and anti-AIDS activities, by the reaction with thiourea (Prabhavat & Ghiya, 1998). A series of similar chalcones is under investigation in our laboratory for their biological activities. We report here on the synthesis and crystal structure of a new chalcone, containing a quinolyl ring system, (2*E*)-3-(2-chloro-6-methylquinolin-3-yl)-1-(naphthalen-1-yl)prop-2-en-1-one.

The title molecule is presented in Fig. 1. The bond distances are as expected (Allen, 2002). The mean planes of the quinoline and naphthalene rings, defined by atoms N1/C1—C9 and C14—C23, respectively, are individually planar with maximum deviations of 0.020 (2) and 0.033 (2) Å, respectively. These planes are inclined at 30.01 (4)° with respect to each other.

The crystal structure is devoid of any classical hydrogen bonds, however in the molecule itself short intramolecular interactions involving atoms Cl1 and O1 are present (Table 1). In the crystal structure a weak C-H···Cl interaction links the molecules to form chains propagating in [001]; see Table 1 and Fig. 2 for details.

### Experimental

2-Chloro-6-methyl-3-formylquinoline was prepared by the literature procedure (Meth-Cohn *et al.*, 1981). A mixture of 2-chloro-6-methyl-3-formylquinoline (2.055 g, 10.0 mmol), 1-acetylnaphthalene (1.7021 g, 10.0 mmol) and methanol (50 ml) was stirred at RT, and an aqueous solution of sodium hydroxide (4.0 ml, 10 %) was added dropwise. The mixture was stirred overnight and was then pored into ice-cold water (200 ml). The precipitates obtained were collected by filtration, washed first with cold water and then with cold methanol. Recrystallization from chloroform gave pale-yellow crystals (yield: 2.90 g; 8.11 mmol, 81.0%), (m.p. 433-435 K).

### Refinement

Although all of the H atoms could be located in the difference Fourier maps they were included at geometrically idealized positions and refined in the riding-model approximation: C—H = 0.95 and 0.98 Å for aromatic and methyl H-atoms, respectively, with *U*_iso_(H) = 1.2*U*_eq_(C).

### Crystal data

**Table:**

C_23_H_16_ClNO	*F*(000) = 744
*M**_r_* = 357.82	*D*_x_ = 1.362 Mg m^−^^3^
Monoclinic, *P*2_1_/*c*	Mo *K*α radiation, λ = 0.71073 Å
Hall symbol: -P 2ybc	Cell parameters from 6886 reflections
*a* = 16.919 (8) Å	θ = 2.8–27.5°
*b* = 7.146 (3) Å	µ = 0.23 mm^−^^1^
*c* = 14.829 (5) Å	*T* = 173 K
β = 103.29 (2)°	Plate, light yellow
*V* = 1744.9 (13) Å^3^	0.14 × 0.12 × 0.05 mm
*Z* = 4	

### Data collection

**Table:**

Nonius KappaCCD diffractometer	3988 independent reflections
Radiation source: fine-focus sealed tube	2575 reflections with *I* > 2σ(*I*)
graphite	*R*_int_ = 0.039
ω and φ scans	θ_max_ = 27.5°, θ_min_ = 2.8°
Absorption correction: multi-scan (*SORTAV*; Blessing, 1997)	*h* = −21→21
*T*_min_ = 0.968, *T*_max_ = 0.989	*k* = −8→9
6886 measured reflections	*l* = −19→19

### Refinement

**Table:**

Refinement on *F*^2^	Primary atom site location: structure-invariant direct methods
Least-squares matrix: full	Secondary atom site location: difference Fourier map
*R*[*F*^2^ > 2σ(*F*^2^)] = 0.051	Hydrogen site location: difference Fourier map
*wR*(*F*^2^) = 0.131	H-atom parameters constrained
*S* = 1.01	*w* = 1/[σ^2^(*F*_o_^2^) + (0.0545*P*)^2^ + 0.6476*P*] where *P* = (*F*_o_^2^ + 2*F*_c_^2^)/3
3988 reflections	(Δ/σ)_max_ < 0.001
236 parameters	Δρ_max_ = 0.24 e Å^−^^3^
0 restraints	Δρ_min_ = −0.29 e Å^−^^3^

### Special details

**Table:**

Geometry. All esds (except the esd in the dihedral angle between two l.s. planes) are estimated using the full covariance matrix. The cell esds are taken into account individually in the estimation of esds in distances, angles and torsion angles; correlations between esds in cell parameters are only used when they are defined by crystal symmetry. An approximate (isotropic) treatment of cell esds is used for estimating esds involving l.s. planes.
Refinement. Refinement of *F*^2^ against ALL reflections. The weighted *R*-factor wR and goodness of fit *S* are based on *F*^2^, conventional *R*-factors *R* are based on *F*, with *F* set to zero for negative *F*^2^. The threshold expression of *F*^2^ > σ(*F*^2^) is used only for calculating *R*-factors(gt) etc. and is not relevant to the choice of reflections for refinement. *R*-factors based on *F*^2^ are statistically about twice as large as those based on *F*, and *R*- factors based on ALL data will be even larger.

### Fractional atomic coordinates and isotropic or equivalent isotropic displacement parameters (Å^2^)

**Table:**

	*x*	*y*	*z*	*U*_iso_*/*U*_eq_	
Cl1	0.05985 (4)	0.69090 (9)	0.33367 (3)	0.0466 (2)	
O1	0.29189 (9)	0.9031 (2)	0.21743 (9)	0.0428 (4)	
N1	−0.07435 (11)	0.6646 (3)	0.20914 (11)	0.0364 (4)	
C1	−0.12197 (12)	0.6652 (3)	0.11997 (13)	0.0319 (5)	
C2	−0.20482 (13)	0.6170 (3)	0.10550 (15)	0.0378 (5)	
H2	−0.2275	0.5874	0.1567	0.045*	
C3	−0.25230 (13)	0.6132 (3)	0.01723 (15)	0.0374 (5)	
H3	−0.3080	0.5801	0.0081	0.045*	
C4	−0.22076 (13)	0.6572 (3)	−0.06116 (14)	0.0353 (5)	
C5	−0.14061 (13)	0.7047 (3)	−0.04742 (14)	0.0344 (5)	
H5	−0.1190	0.7363	−0.0992	0.041*	
C6	−0.08912 (13)	0.7078 (3)	0.04277 (13)	0.0314 (5)	
C7	−0.00604 (13)	0.7522 (3)	0.05985 (14)	0.0338 (5)	
H7	0.0172	0.7835	0.0092	0.041*	
C8	0.04260 (13)	0.7514 (3)	0.14841 (13)	0.0317 (5)	
C9	0.00187 (13)	0.7035 (3)	0.21931 (13)	0.0328 (5)	
C10	−0.27558 (15)	0.6499 (4)	−0.15703 (16)	0.0481 (6)	
H10A	−0.2452	0.6896	−0.2026	0.058*	
H10B	−0.3219	0.7338	−0.1600	0.058*	
H10C	−0.2952	0.5217	−0.1708	0.058*	
C11	0.12863 (13)	0.8006 (3)	0.16855 (13)	0.0340 (5)	
H11	0.1533	0.8344	0.2306	0.041*	
C12	0.17573 (13)	0.8023 (3)	0.10796 (14)	0.0338 (5)	
H12	0.1535	0.7696	0.0452	0.041*	
C13	0.26277 (13)	0.8548 (3)	0.13762 (13)	0.0322 (5)	
C14	0.31294 (12)	0.8425 (3)	0.06743 (13)	0.0295 (5)	
C15	0.27849 (14)	0.8918 (3)	−0.02286 (13)	0.0361 (5)	
H15	0.2231	0.9285	−0.0390	0.043*	
C16	0.32305 (16)	0.8890 (4)	−0.09110 (15)	0.0551 (7)	
H16	0.2983	0.9253	−0.1528	0.066*	
C17	0.40178 (17)	0.8344 (5)	−0.06904 (16)	0.0718 (10)	
H17	0.4316	0.8325	−0.1160	0.086*	
C18	0.44086 (14)	0.7800 (4)	0.02193 (15)	0.0489 (7)	
C19	0.52216 (17)	0.7176 (5)	0.04411 (19)	0.0776 (11)	
H19	0.5520	0.7158	−0.0029	0.093*	
C20	0.55878 (16)	0.6603 (5)	0.13079 (18)	0.0637 (8)	
H20	0.6136	0.6191	0.1443	0.076*	
C21	0.51529 (14)	0.6622 (3)	0.19988 (16)	0.0439 (6)	
H21	0.5407	0.6211	0.2605	0.053*	
C22	0.43669 (13)	0.7222 (3)	0.18163 (14)	0.0361 (5)	
H22	0.4084	0.7225	0.2300	0.043*	
C23	0.39635 (12)	0.7843 (3)	0.09213 (13)	0.0309 (5)	

### Atomic displacement parameters (Å^2^)

**Table:**

	*U*^11^	*U*^22^	*U*^33^	*U*^12^	*U*^13^	*U*^23^
Cl1	0.0431 (4)	0.0673 (4)	0.0265 (3)	−0.0126 (3)	0.0024 (2)	0.0045 (3)
O1	0.0350 (9)	0.0637 (11)	0.0287 (7)	−0.0057 (8)	0.0053 (6)	−0.0084 (7)
N1	0.0347 (11)	0.0459 (11)	0.0292 (9)	−0.0018 (9)	0.0088 (7)	0.0014 (8)
C1	0.0296 (12)	0.0357 (12)	0.0304 (10)	0.0007 (9)	0.0069 (8)	−0.0012 (9)
C2	0.0325 (12)	0.0425 (14)	0.0404 (11)	0.0001 (10)	0.0122 (9)	0.0013 (10)
C3	0.0278 (12)	0.0360 (13)	0.0471 (12)	0.0011 (10)	0.0056 (9)	−0.0001 (10)
C4	0.0336 (12)	0.0302 (12)	0.0387 (11)	0.0032 (9)	0.0014 (9)	−0.0003 (9)
C5	0.0365 (13)	0.0357 (12)	0.0306 (10)	−0.0006 (10)	0.0071 (9)	0.0002 (9)
C6	0.0310 (12)	0.0334 (12)	0.0301 (10)	0.0008 (9)	0.0074 (8)	−0.0015 (9)
C7	0.0340 (12)	0.0409 (13)	0.0282 (10)	−0.0013 (10)	0.0107 (8)	−0.0011 (9)
C8	0.0301 (11)	0.0371 (12)	0.0284 (10)	−0.0013 (9)	0.0081 (8)	−0.0031 (9)
C9	0.0329 (12)	0.0396 (13)	0.0256 (9)	−0.0005 (10)	0.0059 (8)	−0.0007 (9)
C10	0.0419 (14)	0.0500 (15)	0.0448 (13)	−0.0038 (12)	−0.0056 (11)	0.0043 (11)
C11	0.0318 (12)	0.0404 (13)	0.0287 (10)	−0.0021 (10)	0.0043 (8)	−0.0006 (9)
C12	0.0298 (11)	0.0418 (13)	0.0284 (9)	−0.0005 (10)	0.0041 (8)	−0.0034 (9)
C13	0.0297 (12)	0.0363 (12)	0.0294 (10)	−0.0014 (9)	0.0041 (8)	−0.0007 (9)
C14	0.0277 (11)	0.0319 (12)	0.0277 (9)	−0.0013 (9)	0.0039 (8)	−0.0022 (8)
C15	0.0323 (12)	0.0427 (13)	0.0309 (10)	0.0031 (10)	0.0023 (9)	0.0004 (9)
C16	0.0447 (15)	0.093 (2)	0.0267 (11)	0.0093 (14)	0.0061 (10)	0.0086 (12)
C17	0.0466 (17)	0.142 (3)	0.0321 (12)	0.0179 (18)	0.0187 (11)	0.0051 (16)
C18	0.0329 (13)	0.080 (2)	0.0340 (11)	0.0100 (13)	0.0085 (9)	−0.0020 (12)
C19	0.0408 (16)	0.148 (3)	0.0466 (14)	0.0298 (19)	0.0155 (12)	−0.0024 (18)
C20	0.0354 (15)	0.094 (2)	0.0567 (16)	0.0209 (15)	0.0012 (12)	−0.0071 (15)
C21	0.0383 (14)	0.0456 (14)	0.0409 (12)	0.0005 (11)	−0.0051 (10)	0.0013 (11)
C22	0.0321 (12)	0.0404 (13)	0.0335 (11)	−0.0040 (10)	0.0025 (9)	0.0028 (9)
C23	0.0281 (11)	0.0341 (12)	0.0290 (10)	−0.0021 (9)	0.0035 (8)	−0.0033 (9)

### Geometric parameters (Å, °)

**Table:**

Cl1—C9	1.755 (2)	C11—H11	0.9500
O1—C13	1.222 (2)	C12—C13	1.485 (3)
N1—C9	1.294 (3)	C12—H12	0.9500
N1—C1	1.381 (3)	C13—C14	1.489 (3)
C1—C2	1.411 (3)	C14—C15	1.378 (3)
C1—C6	1.416 (3)	C14—C23	1.436 (3)
C2—C3	1.370 (3)	C15—C16	1.394 (3)
C2—H2	0.9500	C15—H15	0.9500
C3—C4	1.421 (3)	C16—C17	1.354 (4)
C3—H3	0.9500	C16—H16	0.9500
C4—C5	1.367 (3)	C17—C18	1.414 (3)
C4—C10	1.509 (3)	C17—H17	0.9500
C5—C6	1.419 (3)	C18—C19	1.411 (4)
C5—H5	0.9500	C18—C23	1.418 (3)
C6—C7	1.406 (3)	C19—C20	1.356 (4)
C7—C8	1.380 (3)	C19—H19	0.9500
C7—H7	0.9500	C20—C21	1.392 (4)
C8—C9	1.425 (3)	C20—H20	0.9500
C8—C11	1.460 (3)	C21—C22	1.364 (3)
C10—H10A	0.9800	C21—H21	0.9500
C10—H10B	0.9800	C22—C23	1.417 (3)
C10—H10C	0.9800	C22—H22	0.9500
C11—C12	1.331 (3)		
			
C9—N1—C1	117.22 (17)	C11—C12—C13	120.61 (18)
N1—C1—C2	119.03 (18)	C11—C12—H12	119.7
N1—C1—C6	121.58 (19)	C13—C12—H12	119.7
C2—C1—C6	119.37 (18)	O1—C13—C12	120.64 (19)
C3—C2—C1	119.6 (2)	O1—C13—C14	121.71 (19)
C3—C2—H2	120.2	C12—C13—C14	117.64 (17)
C1—C2—H2	120.2	C15—C14—C23	119.57 (18)
C2—C3—C4	122.0 (2)	C15—C14—C13	118.85 (19)
C2—C3—H3	119.0	C23—C14—C13	121.57 (17)
C4—C3—H3	119.0	C14—C15—C16	121.6 (2)
C5—C4—C3	118.63 (19)	C14—C15—H15	119.2
C5—C4—C10	121.5 (2)	C16—C15—H15	119.2
C3—C4—C10	119.9 (2)	C17—C16—C15	119.7 (2)
C4—C5—C6	121.2 (2)	C17—C16—H16	120.2
C4—C5—H5	119.4	C15—C16—H16	120.2
C6—C5—H5	119.4	C16—C17—C18	121.9 (2)
C7—C6—C1	117.71 (18)	C16—C17—H17	119.1
C7—C6—C5	123.03 (19)	C18—C17—H17	119.1
C1—C6—C5	119.26 (19)	C19—C18—C17	121.8 (2)
C8—C7—C6	121.54 (19)	C19—C18—C23	119.3 (2)
C8—C7—H7	119.2	C17—C18—C23	118.9 (2)
C6—C7—H7	119.2	C20—C19—C18	121.7 (2)
C7—C8—C9	114.79 (19)	C20—C19—H19	119.2
C7—C8—C11	122.81 (19)	C18—C19—H19	119.2
C9—C8—C11	122.37 (18)	C19—C20—C21	119.4 (2)
N1—C9—C8	127.14 (18)	C19—C20—H20	120.3
N1—C9—Cl1	114.98 (15)	C21—C20—H20	120.3
C8—C9—Cl1	117.87 (16)	C22—C21—C20	120.9 (2)
C4—C10—H10A	109.5	C22—C21—H21	119.6
C4—C10—H10B	109.5	C20—C21—H21	119.6
H10A—C10—H10B	109.5	C21—C22—C23	121.5 (2)
C4—C10—H10C	109.5	C21—C22—H22	119.3
H10A—C10—H10C	109.5	C23—C22—H22	119.3
H10B—C10—H10C	109.5	C22—C23—C18	117.3 (2)
C12—C11—C8	125.99 (19)	C22—C23—C14	124.28 (19)
C12—C11—H11	117.0	C18—C23—C14	118.41 (18)
C8—C11—H11	117.0		
			
C9—N1—C1—C2	177.8 (2)	C11—C12—C13—O1	−2.4 (3)
C9—N1—C1—C6	−0.4 (3)	C11—C12—C13—C14	176.4 (2)
N1—C1—C2—C3	−178.6 (2)	O1—C13—C14—C15	−143.8 (2)
C6—C1—C2—C3	−0.3 (3)	C12—C13—C14—C15	37.4 (3)
C1—C2—C3—C4	−0.3 (3)	O1—C13—C14—C23	34.9 (3)
C2—C3—C4—C5	0.0 (3)	C12—C13—C14—C23	−143.8 (2)
C2—C3—C4—C10	179.7 (2)	C23—C14—C15—C16	−0.9 (3)
C3—C4—C5—C6	0.9 (3)	C13—C14—C15—C16	177.9 (2)
C10—C4—C5—C6	−178.8 (2)	C14—C15—C16—C17	0.9 (4)
N1—C1—C6—C7	−0.8 (3)	C15—C16—C17—C18	−0.2 (5)
C2—C1—C6—C7	−179.0 (2)	C16—C17—C18—C19	178.0 (3)
N1—C1—C6—C5	179.4 (2)	C16—C17—C18—C23	−0.5 (5)
C2—C1—C6—C5	1.2 (3)	C17—C18—C19—C20	−178.0 (3)
C4—C5—C6—C7	178.7 (2)	C23—C18—C19—C20	0.5 (5)
C4—C5—C6—C1	−1.5 (3)	C18—C19—C20—C21	0.2 (5)
C1—C6—C7—C8	1.1 (3)	C19—C20—C21—C22	−0.5 (5)
C5—C6—C7—C8	−179.1 (2)	C20—C21—C22—C23	0.1 (4)
C6—C7—C8—C9	−0.3 (3)	C21—C22—C23—C18	0.5 (3)
C6—C7—C8—C11	−178.7 (2)	C21—C22—C23—C14	177.5 (2)
C1—N1—C9—C8	1.5 (3)	C19—C18—C23—C22	−0.8 (4)
C1—N1—C9—Cl1	−177.48 (16)	C17—C18—C23—C22	177.7 (3)
C7—C8—C9—N1	−1.1 (3)	C19—C18—C23—C14	−178.0 (3)
C11—C8—C9—N1	177.3 (2)	C17—C18—C23—C14	0.5 (4)
C7—C8—C9—Cl1	177.80 (17)	C15—C14—C23—C22	−176.9 (2)
C11—C8—C9—Cl1	−3.7 (3)	C13—C14—C23—C22	4.4 (3)
C7—C8—C11—C12	−19.2 (4)	C15—C14—C23—C18	0.1 (3)
C9—C8—C11—C12	162.5 (2)	C13—C14—C23—C18	−178.6 (2)
C8—C11—C12—C13	−179.8 (2)		

### Hydrogen-bond geometry (Å, °)

**Table:**

*D*—H···*A*	*D*—H	H···*A*	*D*···*A*	*D*—H···*A*
C7—H7···Cl1^i^	0.95	2.86	3.792 (2)	166
C11—H11···Cl1	0.95	2.65	3.045 (2)	106
C11—H11···O1	0.95	2.45	2.788 (3)	101
C22—H22···O1	0.95	2.33	2.924 (3)	120

Symmetry codes: (i) *x*, −*y*+3/2, *z*−1/2.
